# A lymphoepithelial cyst in the pancreatic accessory spleen: A case report

**DOI:** 10.1002/ccr3.4241

**Published:** 2021-06-22

**Authors:** Sawako Hiroi, Michinori Hamaoka, Rie Yamamoto, Yasuhiro Matsugu, Takashi Nishisaka, Hideki Nakahara, Toshiyuki Itamoto

**Affiliations:** ^1^ Department of Gastroenterological Surgery Hiroshima Prefectural Hospital Hiroshima Japan; ^2^ Department of Pathology Hiroshima Prefectural Hospital Hiroshima Japan

**Keywords:** a lymphoepithelial cyst, pancreatic accessory spleen

## Abstract

We present the first report of a lymphoepithelial cyst. As additional cases will likely be encountered in the future, our study sets the precedent for future research.

## Graphical abstract

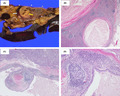

## 1 INTRODUCTION

There are 57 reports of epidermoid cysts in the accessory spleen in the pancreas, but none of lymphoepithelial cysts (LECs) in this location. The pathological diagnostic criteria for LECs are still unclear and controversial. We describe an LEC in the accessory spleen, including the pathological findings.

Pancreatic cysts are broadly categorized as neoplastic or non‐neoplastic. The former category includes intraductal papillary mucinous neoplasms (IPMNs), mucinous cystic neoplasms (MCNs), and serous cystic neoplasms (SCNs), and the latter includes pancreatic pseudocysts and lymphoepithelial, epidermoid, and dermoid cysts.^1^ There are also pancreatic neuroendocrine tumors (pNETs), which are tumors that have degenerated into cysts. These classifications are laid out in several documents including the International Association of Pancreatology guidelines,^2^ the European expert consensus statements,^3^ and the American Gastroenterological Association guidelines.^4^ However, there are no guidelines or clear diagnostic criteria for pancreatic pseudocysts.

Pancreatic lymphoepithelial cysts (LECs) are rare benign lesions initially described by Luchtrath and Schriefers in 1985^5^ and named by Truong et al in 1987.^6^ LECs are typically observed in middle‐aged and elderly men; they occur equally in the pancreatic head, body, or tail, and may present as a single or multilocular lesion. This study is the first to document a case of an LEC in the pancreatic accessory spleen, and thus, in this respect, we believe it is valuable. Furthermore, the knowledge acquired in this study will enable the development of international diagnostic criteria for LECs and similar epidermoid cysts and dermoid cysts in the future.

## 2 PRESENTATION OF THE CASE

A 43‐year‐old man was found to have a pancreatic tail cyst on abdominal ultrasonography at a previous hospital. He had no subjective symptoms or medical history. Laboratory examinations showed normal results for the following: complete blood cell counts, hepatic and renal function, and serum levels of carcinoembryonic antigen (CEA), carbohydrate antigen 19‐9 (CA 19‐9), amylase, lipase, and glucose.

**FIGURE 1:**
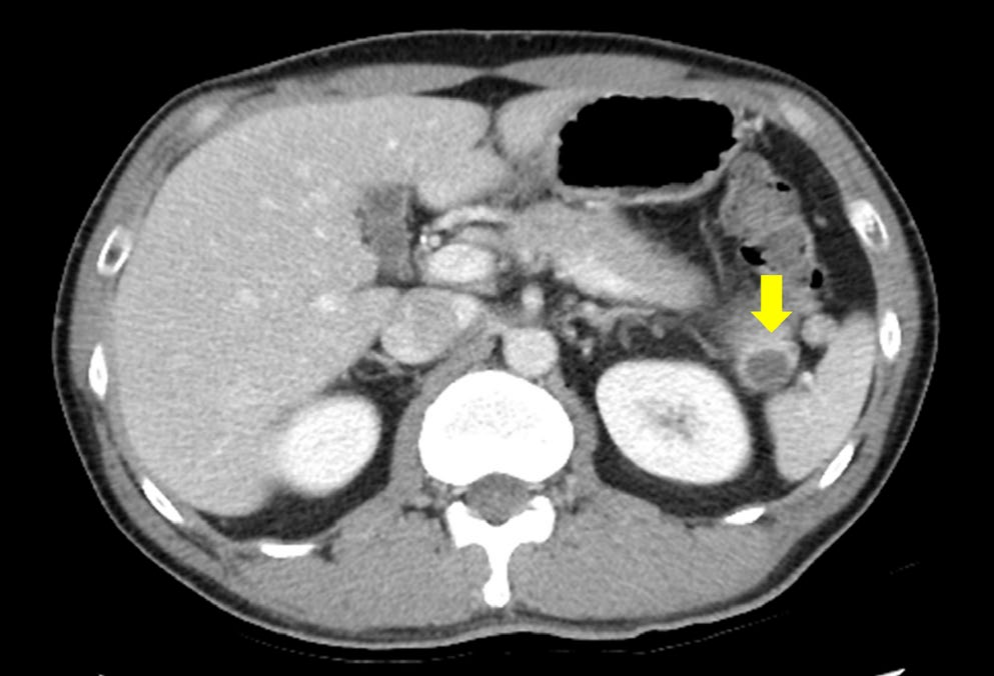
Computed tomography (CT): A cystic mass with a diameter of 15 mm in the pancreatic tail (arrow) is shown

**FIGURE 2:**
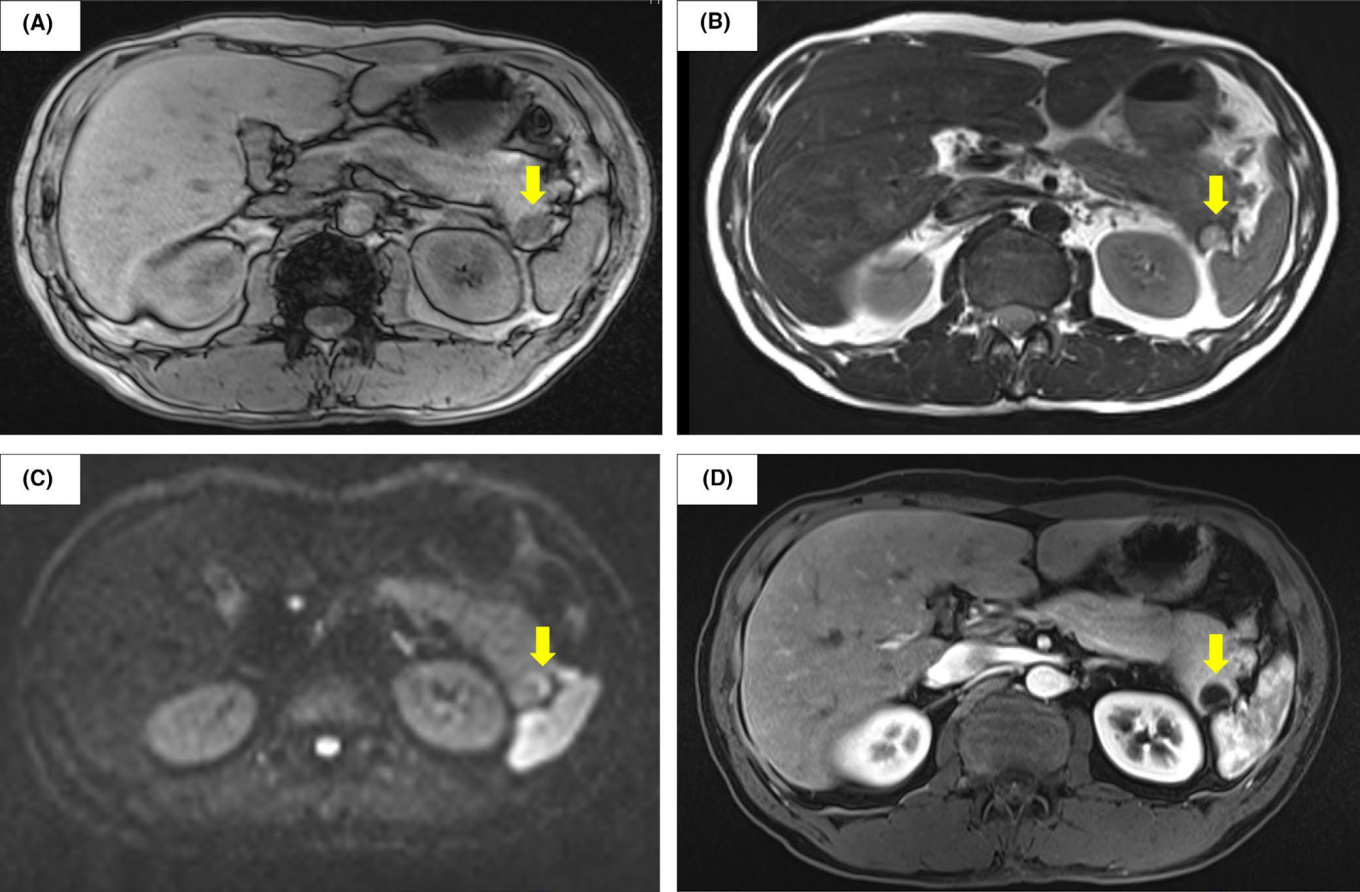
Magnetic resonance imaging (MRI): A, The T1‐weighted image shows a cystic lesion measuring 18 × 14 mm with low signal intensity (arrow). B, The same lesion has a high signal intensity on a T2‐weighted image (arrow). C, Part of the cyst wall was hyperintense (arrow) on diffusion‐weighted imaging. D, Early uptake of the dye by this part of the wall (arrow) on dynamic MRI is shown

**FIGURE 3:**
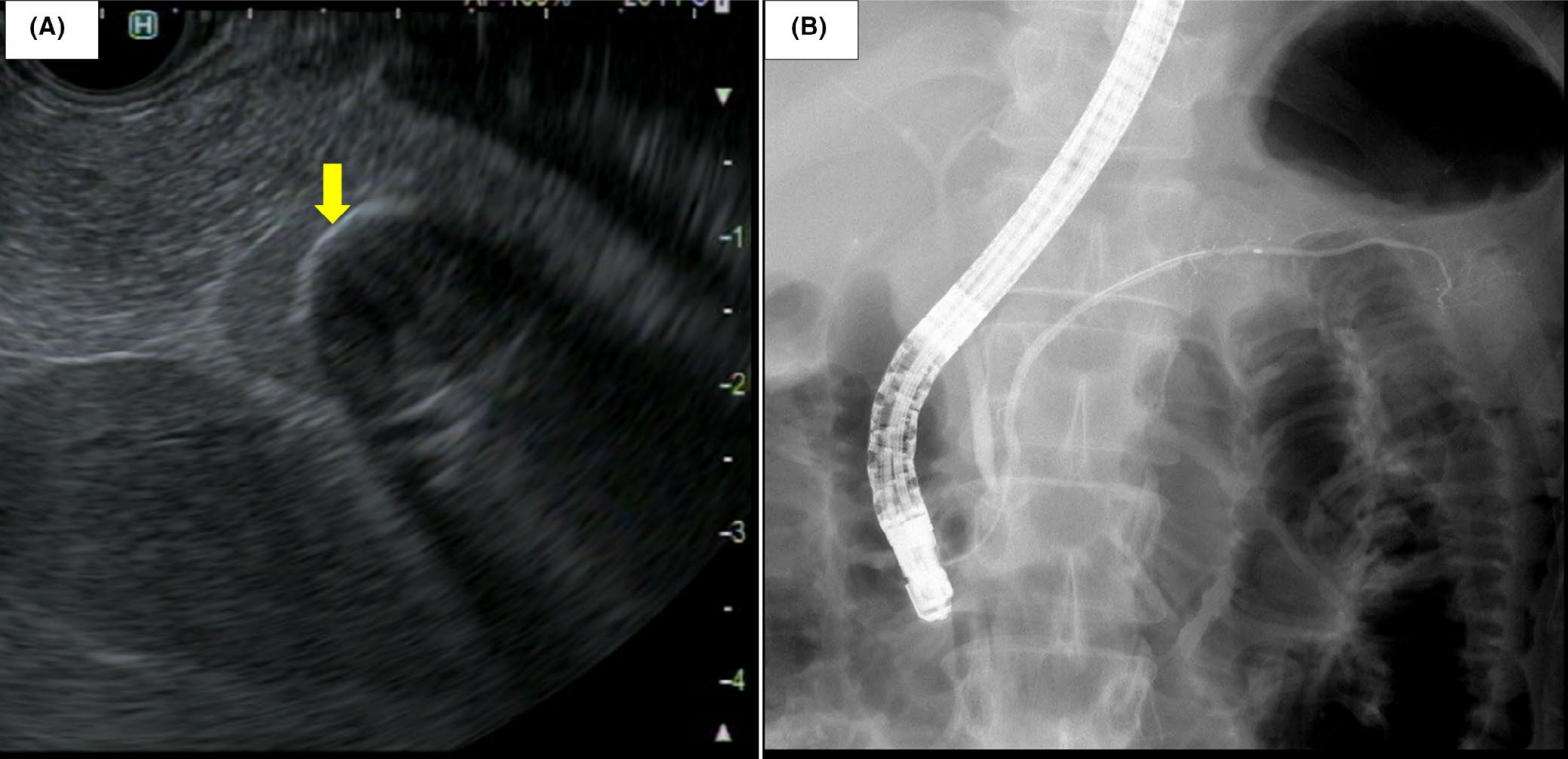
Endoscopic imaging: A, Endoscopic ultrasonography (EUS) shows a cyst with calcification in the margin (arrow). B, Endoscopic retrograde cholangiopancreatography shows a normal main pancreatic duct with no communication with the cystic lesion

The patient was referred to our hospital, and the presence of the cyst in the pancreatic tail was confirmed via computed tomography (CT) (Figure 1). The cystic lesion was 15 cm in diameter, monocystic, and surrounded by an area of low intensity. Magnetic resonance imaging (MRI) revealed a lesion with depleted and enhanced intensity on T1‐ and T2‐weighted images, respectively (Figure 2A, B). Diffusion‐weighted MRI displayed increased signal potency in the peripheral portion of the cystic lesion (the wall and the septa) (Figure 2C); the cystic contents had reduced intensity on enhanced MRI (Figure 2D). Additional observations included a cyst with high‐echoic lesions, a pancreatic tail, and calcification on the cyst margin on endoscopic ultrasonography (Figure 3A), and a slight displacement of the pressure superior to the duct with no narrowing or disruption on endoscopic retrograde cholangiopancreatography (Figure 3B).

The CT and T1‐ and T2‐weighted MRI data suggested cyst degeneration, which is a characteristic of pNETs. Hence, the preoperative diagnosis was a pNET. Distal pancreatectomy was performed with concomitant splenectomy; the guidelines mentioned above recommend surgery as the first‐line treatment for pNETs.

**FIGURE 4:**
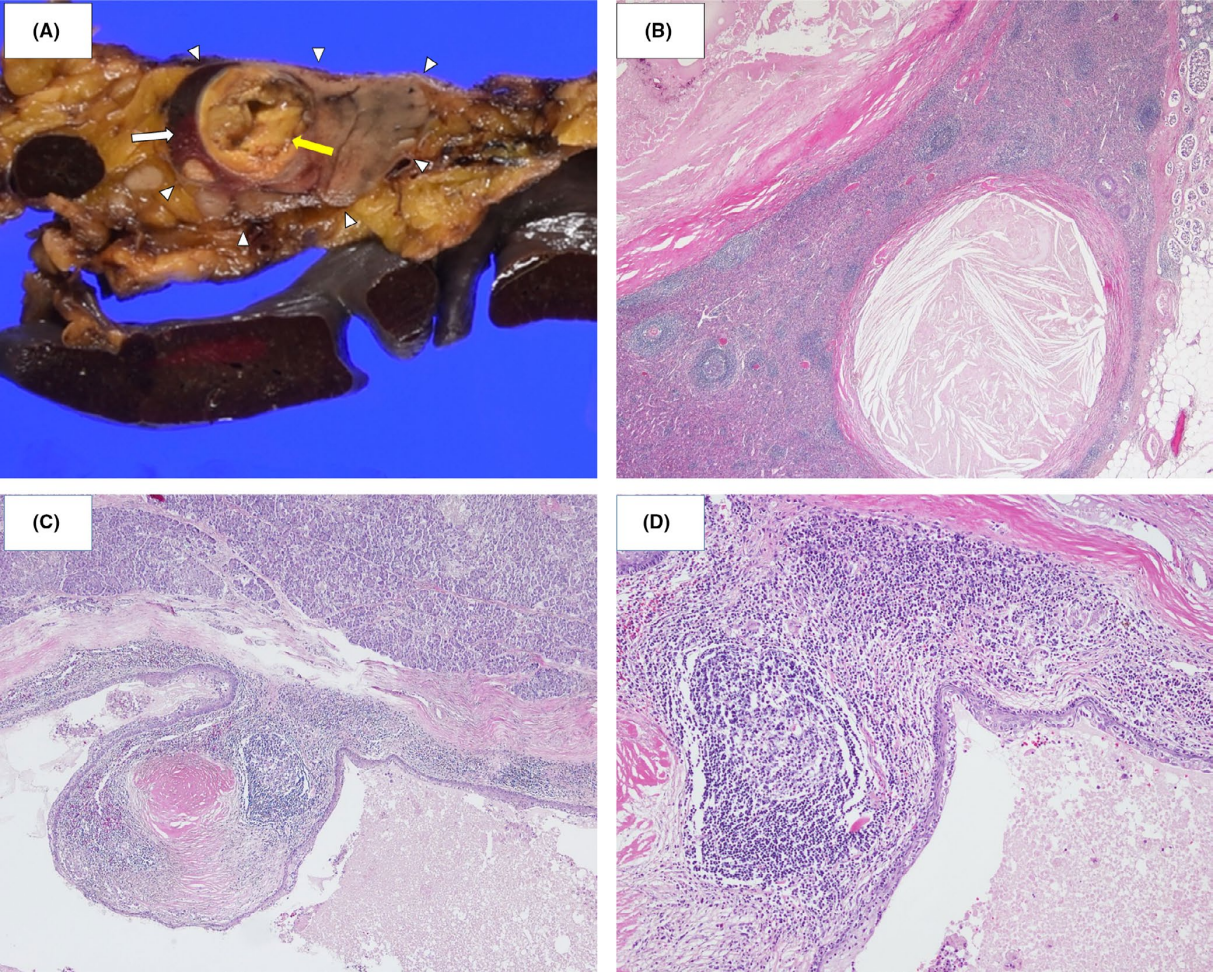
Macroscopic findings and histopathology: A, The pancreatic parenchyma at the tail of the pancreas (white arrowhead) contains spleen tissue (white arrow) and a multilocular cyst with a maximum diameter of 17 mm (yellow arrow). B, The cystic wall is lined by mature keratinized squamous epithelium and underlying lymphoid tissue (×20 magnification, hematoxylin and eosin staining). C, The epithelial lining is surrounded by splenic pulp and pancreatic tissue (×40 magnification, hematoxylin and eosin staining). D, Lymphoid follicles and lymphoid tissue are observed (×100 magnification, hematoxylin and eosin staining)

The postoperative course was uneventful. The excised surface of a resected specimen indicated a multilocular cyst with solid nodules (Figure 4A). Histopathological examination revealed the presence of spleen tissue, both red and white pulp, in the parenchyma of the pancreatic tail. Hence, we diagnosed the tumor as an intrapancreatic accessory spleen cyst, with the tumor originating in the accessory spleen rather than in the tissue between the tail of the pancreas and the spleen. The major and minor multilocular cysts had a maximum diameter of 17 mm. The luminal epithelium consisted of mature squamous epithelium and subepithelial lymphoid tissue (Figure 4B), and the cyst lumen contained keratin and cholesterol deposits in the clefts. The lymphatic tissue occupied the majority of the cyst (Figure 4D), which is a diagnostic criterion for LECs. Ultimately, the patient was diagnosed with an LEC of the pancreatic accessory spleen.

## 3 DISCUSSION

An accessory spleen is not uncommon. Halpert et al reported 291 (10.8%) accessory spleens in 2700 autopsied cases; 215 (62.1%) were in the vicinity of the splenic hilum, followed by 78 (22.5%) in the pancreatic tail.^7^ However, a cyst occurring in a pancreatic accessory spleen is extremely rare, with only 57 reported cases (Table 1).^8^, ^9^, ^10^, ^11^, ^12^, ^13^, ^14^, ^15^, ^16^, ^17^, ^18^, ^19^, ^20^, ^21^, ^22^, ^23^, ^24^, ^25^, ^26^, ^27^, ^28^, ^29^, ^30^, ^31^, ^32^, ^33^, ^34^, ^35^, ^36^, ^37^, ^38^, ^39^, ^40^, ^41^, ^42^, ^43^, ^44^, ^45^, ^46^, ^47^, ^48^, ^49^, ^50^, ^51^, ^52^, ^53^ The lesion site was the pancreatic tail in all 57 cases. The primary complaints were abdominal pain and vomiting, although 33 of the 57 cases were asymptomatic. Tumors with smaller diameters are often asymptomatic.

**TABLE 1:** English language reports of cysts in the accessory spleen in the pancreas

Case	Authors	Sex/age	Symptom	Location	Size(cm)	Cyst	Serum markers CEA CA19‐9	CT	MRI	Preoperative diagnosis	Surgery	Pathology	Final diagnosis
1	Davidson	M/40	Nausea	Tale	5.5	Multilocular	NI	Cystic lesion surrounded by thin rim of tissue	NI	Pseudocyst, cystadenoma, and cystadenocarcinoma	DP	Cyst wall focally shows a transition from low cuboidal to low stratified squamous epithelium. A giant‐cell granulomatous reaction in the underlying red pulp is surrounded by a band of fibrous tissue like that beneath the epithelium of the cyst wall	Epidermoid cyst
2	Hanada	M/51	Abdominal pain	Tail	6	NI	NI	Cystic mass with a rim of dense density	NI	Pseudocyst	DP	The wall of the cyst was composed of dense, hypocellular, collagenous tissue. The inner surface of the cyst was lined by a flattened stratified squamous type of epithelium	Epidermoid cyst
3	Morohoshi	F/32	Abdominal pain	Tail	6	Unilocular	Normal	Well‐demarcated cystic lesion	NI	Pancreatic cyst	Cyst removal	The cystic wall consisted of three tissue elements: The inside was lined by stratified epithelium, the middle layer was composed of some splenic pulp, and the peripheral layer consisted of dense fibrous connective tissue containing some involutional pancreatic ducts and islets. The lining epithelium was mature stratified squamous epithelium	Epidermoid cyst
4	Nakae	F/37	Epigastric pain	Tail	6.5	Unilocular	NI	Cystic lesion with a thin wall of high density	T1 low, T2 high	Pancreatic cyst	SPDP	The cyst is surrounded with fibrous tissue and a thin layer of splenic tissue containing a germinal center, adjacent to normal pancreatic tissue	Epidermoid cyst
5	Tang	M/38	Asymptomatic	Tail	1.4	Multilocular	NI	Well‐demarcated hypodense lesion	NI	NI	DP	The cysts are lined by nonkeratinizing, stratified squamous epithelium. Mucic‐containing cells are scattered among the epithelium	Epidermoid cyst
6	Furukawa	M/45	Asymptomatic	Tail	2	Multilocular	NI	Peripherally enhanced area, its density is equal to the spleen	NI	Primary cystic neoplasm	DP	The cyst is lined by stratified squamous epithelium. Cyst is present within normal splenic tissue, which is surrounded by pancreatic parenchyma.	Epidermoid cyst
7	Higaki	F/46	Left back pain	Tail	3	Multilocular	CA19‐9:high, 201 U/mL	Oval nodule with a distinct margin	NI	Malignant tumor	DP	Cyst surrounded by accessory splenic tissue The cyst wall was lined with stratified squamous epithelium	Epidermoid cyst
8	Tateyama	F/67	Abdominal pain	Tail	3	Multilocular		Cystic mass of low density	NI	NI	DP	The cyst is lined by stratified squamous epithelium. The epithelial lining is surrounded by hyalinized fibrous tissue with scattered lymphoid tissue, splenic pulp, and pancreatic tissue	Epidermoid cyst
9	Sasou	F/49	Asymptomatic	Tail	4.3	Multilocular	NI	NI	NI	Pancreatic cystic tumor	DP	The cyst is surrounded by splenic tissue in the pancreas. The inside of the cyst in the accessory spleen was lined with stratified squamous epithelium and a layer of flat cells	Epithelial cyst
10	Choi	F/54	Epigastric pain	Tail	15	Multilocular	NI	Major cystic component, small solid component with the same homogeneous	Cyst:T1 low, T2 high:solid lesion: T1 low, T2 intermediate‐high	Benign cyst of the pancreas or accessory spleen	DP	The cyst is lined by stratified squamous epithelium with keratinization. A thin fibrous capsule separates the intrapancreatic accessory spleen from the pancreas	Epidermoid cyst
11	Tsutumi	M/51	Asymptomatic	Tail	2.5	Multilocular	Normal	Well‐demarcated cystic lesion containing a solid portion	Cystic lesion containing a solid	Benign cyst of the pancreas	DP	Cystic lesions are seen in the splenic tissue surrounded by pancreatic tissue. Cystic walls are lined by stratified squamous epithelium	Epidermoid cyst
12	Horibe	M/48	Asymptomatic	Tail	2	Unilocular	CA19‐9:high 53 U/mL	No substance in the cyst by enhanced image	NI	Mucin‐producing pancreatic tumor	DP	The cyst is surrounded with fibrous tissue and a thin layer of splenic tissue containing a germinal center, adjacent to normal pancreatic tissue. The cyst wall is lined with stratified and a few layers of squamous epithelium	Epidermoid cyst
13	Sonomura	F/45	Epigastric pain	Tail	3.5	Multilocular	NI	Parenchymal medial lesion with calcification and cystic lateral lesion	NI	Cystadenoma or solid tumor of the pancreas	DP	The parenchymal region of the mass showed historic features of the spleen, and the multilocular cyst was lined with several layers of stratified squamous epithelium	Epidermoid cyst
14	Fink	F/12	Fever	Tail	10	Multilocular	NI	Rim enhancing cystic lesion, with a medial mural nodule	NI	Infected abdominal cyst	Cyst removal	The cyst was surrounded by nonkeratinizing squamous epithelium. The wall was composed of fibrous tissue with prominent hemosiderosis surrounded by splenic tissue. The pancreas was compressed by the splenic capsule	Epidermoid cyst
15	Yokimizo	M/38	Asymptomatic	Tail	3	Multilocular	CA19‐9:high 410 U/mL	NI	Cyst:T2 super‐high, Cyst wall: delineated enhancement	MCN, adenocarcinoma and ECIPS	DP	The cyst wall was lined by nonkeratinizing stratified squamous epithelium and containing splenic tissue	Epidermoid cyst
16	Kanazawa	F/58	Asymptomatic	Tail	2.5	Multilocular	CA19‐9:high 62 U/mL	Septated low‐density area	Cystic component: T1 hypo, T2 hyper	MCN	SPDP	The cyst was lined with stratified squamous epithelium and was surrounded by normal splenic tissue	Epidermoid cyst
17	Watanabe	F/55	Posyprandialepogastralgia	Tail	2.5	Multilocular	CA19‐9:high 176 U/mL	Multilocular cystic tumor. No protruded lesion in the inner lumen	T1 low, T2 high	Mucinous cystadenoma and cystadenocarcinoma	DP	The cystic lesions were surrounded by fibrous tissue and a thin layer of splenic tissue with a germinal center and separated from the adjacent pancreatic parenchyma	Epidermoid cyst
18	Won	M/32	Asymptomatic	Tail	7.5	Unilocular	CA19‐9:high 53 U/mL	Well‐circumscribed cystic mass with inner fluid debris or hemorrhagic fluid	NI	Pancreatic pseudocyst	SPDP	The cyst was surrounded by splenic parenchyma with was lined by nonkeratinizing squamous epithelium and flattened or cuboidal epithelial cells that continued to the stratified squamous epithelium	Epithelial cyst
19	Won	F/49	Abdominal pain	Tail	2	Multilocular	Normal	Well‐circumscribed cystic tumor septation	NI	Serous or mucinous cystadenoma	Laparoscopic DP	The epithelial lining showed a mixture of flattened mesothelial‐like cells, ciliated cuboidal cells, and stratified squamous epithelial cells. Red pulps in the cystic walls were identifiable	Epithelial cyst
20	Ru	M/41	Asymptomatic	Tail	2.5	Unilocular	NI	Well‐circumscribed tumor which partially compressed the spleen	NI	Cystic lesion of the pancreas	DP	The epithelial lining appeared focally stratified without atypia. Scattered mucinous cells were identified occasionally. A fibrotic band with sclerosis lay underneath the epithelial lining, and overlying spleen tissue	Epidermoid cyst
21	Itano	M/40	Asymptomatic	Tail	4	Unilocular	Normal	Solid component with the same homogeneous attenuation as the spleen	Cyst:T1/T2high;solid component:T1 intermediate‐low	ECIPAS	DP	The cyst was lined with stratified squamous epithelium and was surrounded by normal splenic tissue	Epidermoid cyst
22	Servais	F/52	Asymptomatic	Tail	11.5	Multilocular	CA19‐9/CEA:high	Cystic mass which was thin walled and contained single	NI	Malignant pancreatic neoplasm	DP	Epithelium‐lined cyst with a dense hyalinized fibrous wall surrounded by a normal rim of native pancreatic tissue. The cyst wall demonstrated splenic pulp tissue	Epidermoid cyst
23	Gleeson	F/32	Abdominal pain	Tail	1.5	Unilocular	NI	Demarcated cyst without septation calcification and satellite lesion	NI	Pancreatic cystic neoplasm	DP	The epithelial cyst lining at the top, with surrounding splenic tissue inferior to it, and adjacent pancreas demonstrating chronic pancreatitis inferior to the splenic tissue	Epidermoid cyst
24	Zhang and Wang	F/26	Asymptomatic	Tail	2.5	Unilocular	Normal	Cystic wall revealed a density similar to that of the pancreas	NI	Primary MCN	SPDP	The cyst contained homogenous eosinophilic fluid and was lined with stratified squamous epithelium. Accessory spleen tissue was found under the epithelium and surrounded by a complete fibrous capsule	Epidermoid cyst
25	Reiss	M/49	Asymptomatic	Tail	3.6	Multilocular	NI	Heterogeneously enhancing mass	NI	MCN	DP	The lesion revealed an intrapancreatic accessory spleen lined with stratified squamous epithelium with occasional goblet cells	Epidermoid cyst
26	Kodota	F/57	Asymptomatic	Tail	6	Multilocular	Normal	Cystic wall : a partial enhancement	NI	Pancreatic cystic tumor	DP	The cyst is surrounded by normal splenic tissue and hyalinized fibrous tissue consisting of spleen tissue. The cystic walls were lined with nonkeratinizing stratified squamous epithelium and focally cuboidal epithelium	Epidermoid cyst
27	Kodota	F/70	Asymptomatic	Tail	1.7	NI	CA19‐9:high 48 U/mL CEA: normal	Cystic mass lesion	NI	MCN	DP	The cysts are surrounded by normal splenic tissue and hyalinized fibrous tissue. The lining epithelium shows a nonkeratinizing stratified squamous epithelium	Epidermoid cyst
28	Kodota	M/37	Asymptomatic	Tail	10	NI	CA‐19‐9:high 647 U/mL CEA: normal	Cystic mass lesion with a partial enhancement of the cystic wall	NI	Serous cystic tumor or lymphoepithelial cyst	DP	The cysts are lined with the keratinizing stratified squamous epithelium and focally several layers of the cubical epithelium. The cuboidal epithelium revealed transitional findings to the stratified squamous epithelium	Epidermoid cyst
29	Itano	M/67	Epigastric pain	Tail	1.5	Unilocular	CA19‐9:high:182 U/mL	Cystic tissue and smooth solid component	Cyst:T1 intermediate, T2 high. Solid lesion:T1 intermediate	ECIPAS	Laparoscopic DP	The cyst was surrounded by ectopic splenic tissue with a normal appearance and atrophic pancreatic tissue. The cyst was lined with a stratified squamous epithelium	Epidermoid cyst
30	N.Panagiotopoulos	M/51	No	Tail	2.3	NI	Normal	Well‐defined low attenuation lesion arising exophytically from the tail of the pancreas	NI	potential pancreatic malignancy	DP	The cyst was lined by nonkeratinising stratified squamous epithelium. The cyst was revealed within accessory splenic tissue	Epithelial cyst
31	Horn and Lele	M/62	Abdominal pain	Tail	4.8	Multilocular	NI	Left‐sided retroperitoneal mass with a possible cystic component	NI	NI	DP	The cyst was revealed within accessory splenic tissue. The cysts were lined by stratified squamous epithelium	Epidermoid cyst
32	Iwasaki	F/36	Asymptomatic	Tail	3.4	Unilocular	CA19‐9:high 79 U/mL	Septate low‐density lesion, with an area showing higher degree of enhancement than the pancreas	NI	MCN	Laparoscopic DP	Cystic lesion lined with stratified squamous epithelium and surrounded by an intrapancreatic accessory spleen	Epidermoid cyst
33	Yamanishi	F/55	Asymptomatic	Tail	2.5	Unilocular	CA19‐9: high 90 U/mL	Cyst wall was relatively thick, but not enhanced	Cyst: T1 slightly high, thick, but not enhanced	MCN	DP	The cyst was surrounded by a dense, hyalinized fibrous and a thin layer of splenic tissue	Epidermoid cyst
34	Urakami	F/50	Asymptomatic	Tail	3	Unilocular	NI	Single cyst with a contrasted mass beside it	Cyst: T1 low, T2 high	ECIPAS	Laparoscopic DP	The cyst was surrounded by fibrous tissue and a thin layer of splenic with a general center, adjacent to normal pancreatic parenchyma	Epidermoid cyst
35	Khashab	F/49	Abdominal pain	Tail	2.3	Unilocular	NI	Solid	NI	PNET	Laparoscopic DP	The mass had a well‐defined capsule within which was splenic parenchyma and a small cyst lined by a layer of benign squamous epithelium	Epidermoid cyst
36	Harris	F/39	Asymptomatic	Tail	2.5	NI	NI	Stable hypodense lesion	Pancreatic cystic neoplasm	Malignant cystic tumor	Laparoscopic SPDP	The cyst was surrounded by accessory splenic tissue in the pancreas parenchyma. The cyst was lined by multilayered cuboidal epithelium	Epidermoid cyst
37	Hong	F/54	Abdominal discomfort	Tail	2	Multilocular	NI	Cystic mass	NI	NI	SPDP	A cyst is surrounded by accessory splenic tissue in the pancreas parenchyma. A cyst is lined by multilayered cuboidal epithelium	Epidermoid cyst
38	Hamidian	F/36	Asymptomatic	Tail	5	Multilocular	NI	Cystic lesion	NI	NI	DP	pancreas tissue, spleen tissue, fibrous capsule	Epidermoid cyst
39	Zavras	F/63	Nausea and vomiting	Tail	12.6	NI	CA19‐9:high 5000 U/mL CEA: high 180.4 ng/mL	Mass lesion with solid and cystic components	NI	Malignant tumor of the pancreas	DP	The cyst was lined with multilayered flattened epithelium, reminiscent of squamous epithelium above a red pulp splenic parenchyma	Epidermoid cyst
40	Kumamoto	M/39	Diarrhea	Tail	3.8	NI	CA19‐9:high 286 U/mL	A cyst lesion, surrounded by a crescent‐like solid component with the same	Typical findings of an intrapancreatic accessory spleen	ECIPAS	Laparoscopic SPDP	The cyst was lined by nonkeratinizing stratified squamous epithelium and a few layers of atrophic squamous epithelium. The outside cystic wall was composed of relatively thick fibrous connective tissues. The brown solid component was composed of both red and white pulp, locating in the pancreatic parenchyma	Epidermoid cyst
41	Kwak	F/21	Abdominal pain andfever	Tail	2.5	Multilocular	Normal	The wall of the cyst was relatively regular, thick and enhanced	Cyst:T1 iso, T2 hyper. Rim showed hyperintensity in DWI	SPT	Laparoscopic DP	The cyst was lined by stratified squamous epithelium within splenic parenchyma	Epidermoid cyst
42	Kato	F/33	Asymptomatic	Tail	3	Multilocular	Normal	The densities of the solid component and spleen on enhanced CT were similar	The intensity of the solid component T1 and T2 was similar to that of the spleen	SPT and NET	Laparoscopic SPDP	The solid component included splenic tissue with typical red and white pulp. The cyst was lined with a multilayered of nonkeratinized stratified squamous epithelium without any skin appendage, and the squamous epithelium was covered with a hobnail‐like growth epithelium	Epidermoid cyst
43	Modi	F/62	Abdiminal pain	Tail	2.4	Unilocular	NI	Cystic lesion	NI	NI	Laparoscopic DP	The cyst within an intrapancreatic accessory spleen showing a thin epithelial layer with ectopic splenic tissue	Epidermoid cyst
44	Fujii	F/50	Asymptomatic	Tail	4	Unilocular	CA19‐9: high 43.1 U/mL	A unilocular cystic lesion with same enhancement as the adjacent spleen	T1 low/T2 high	MCN	Laparoscopic SPDP	The cyst wall showed a thin multilayered squamous epithelium, with small patches of splenic epithelium	Epidermoid cyst
45	Fujii	F/60	Back discomfort	Tail	3.5	Multilocular	Ca:19‐9: high 52.9 U/mL	A multilocular cystic lesion, solid component with enhancement similar to the spleen	Low T1 and high T2	IPMN	Laparoscopic DP	The cyst wall showed a thin multilayered squamous epithelium, with small patches of splenic epithelium	Epidermoid cyst
46	Guo‐Dong Shan	M/39	Epigastric pain	Tail	3.5	NI	Normal	A cystic lesion in tail of pancreas	Low T1 and high T2	Pancreatic cystadenoma	DP	Old hemorrhage in spleen tissue and formation of capsule wall, surrounded by pancreatic tissue	Hematoma
47	Anghela Paredes	F/17	Abdominal pain, nausea,vomiting	Tail	4.3	NI	Normal	A cystic lesion in tail of pancreas	NI	MCN,IPMN	Laparoscopic SPDP	A cyst was lined by squamous epithelium arising in an accessory spleen	Epidermoid cyst
48	Hirabayashi	M/38	Asymptomatic	Tail	3	Multilocular	Normal	NI	NI	NI	DP	cyst in intrapancreatic accessory spleens are lined by stratified squamous or urothelial epithelium	Epidermoid cyst
49	Hirabayashi	F/40	Abdominal pain	Tail	3.5	Multilocular	CA19‐9: high, 198.7 U/mL	NI	NI	NI	Enucleation	Cyst in intrapancreatic accessory spleens are lined by stratified squamous or urothelial epithelium	Epidermoid cyst
50	Hirabayashi	F/39	Asymptomatic	Tail	2	Multilocular	CA19.9:high,31.9 U/mL	NI	NI	NI	DP	cyst in intrapancreatic accessory spleens are lined by stratified squamous or urothelial epithelium	Epidermoid cyst
51	Hirabayashi	M/54	Asymptomatic	Tail	2.7	Multilocular	Normal	NI	NI	NI	Enucleation	cyst in intrapancreatic accessory spleens are lined by stratified squamous or urothelial epithelium	Epidermoid cyst
52	Hirabayashi	M/55	Asymptomatic	Tail	3.5	Multilocular	CA19‐9: high,50.6 U/mL	NI	NI	NI	Enucleation	Cyst in intrapancreatic accessory spleens are lined by stratified squamous or urothelial epithelium	Epidermoid cyst
53	Hirabayashi	M/36	Asymptomatic	Tail	13.4	Multilocular	CA19‐9: high, 47.2 U/mL	NI	NI	NI	DP	Cyst in intrapancreatic accessory spleens are lined by stratified squamous or urothelial epithelium	Epidermoid cyst
54	Matsumoto	F/40	Asymptomatic	Tail	1.5	Multilocular	Normal	A multilocular cystic lesion, solid periphery, with the same enhancement as the spleen	High T1 and T2 weighted images	ECIPAS	No	Splenic endothelial cells formed sinusoids and abundant polymorphous lymphocytes	Epidermoid cyst
55	Bo Zhou	M/32	Asymptomatic	Tail	3.5	Multilocular	Normal	A well‐defined cystic neoplasm without enhancing mural nodes	NI	MCN	Laparoscopic SPDP	cyst surrounded by accessory splenic tissue in the pancreas parenchyma and cyst wall showed a thin multilayered squamous epithelium	Epidermoid cyst
56	Takagi	M/73	Asymptomatic	Tail	2.4	Multilocular	CA19‐9: high,901 U/mL	A cystic mass	Low T1 and varying intensities T2‐weighted	Malignant tumor of the pancreas	DP	The lesion was located within pancreatic parenchyma. The lesion comprised an accessory spleen and multiple cysts containing mucinous material. Most of the cystic wall were lined by stratified squamous epithelium	Epidermoid cyst
57	Current	M/43	Asymptomatic	Tail	1.5	Multilocular	Normal	A cystic lesion in tail of pancreas	High T1 and T2 weighted images	pNET	Laparoscopic SPDP	The cystic wall is lined by mature keratinized squamous epithelium and underlying lymphoid tissue. The epithelial lining is surrounded by splenic pulp and pancreatic tissue	Lymphoepithelial cyst in accessory spleen

The cyst in the accessory spleen in our study was 1.5 cm in diameter, making it the smallest of those previously reported (range, 15‐134 mm), and apparently had no symptoms. Levels of CEA or CA 19‐9 were elevated in some previous cases, but within normal limits in others. In many cases, the lesion was asymptomatic and detected by imaging. Cyst morphology was multilocular in 33 cases and unilocular in 15. Most cysts had low intensity on T1‐weighted MRI and high intensity on T2‐weighted MRI. Preoperative diagnoses, such as an IPMN, pNET, epidermoid cyst, and malignant tumor, suggest case complexity. The preoperative diagnosis in our case was a pNET, whereas the histopathologically confirmed diagnosis was an LEC in the accessory spleen. This is the first report of an LEC in this location.

Pancreatic LECs are extremely rare, accounting for only 0.5% of pancreatic cysts. Mege et al examined pancreatic LECs in 91 middle‐aged to elderly men (mean age, 55 years; range, 20‐82 years); the lesion was occasionally accompanied by abdominal pain (43%) and an elevated serum CA 19‐9 level (55%).^54^ Pancreatic cysts are classified as true cysts, pseudocysts, or cystic neoplasms. LEC is considered to be a type of true cyst, with a lining of squamous epithelium and dense subepithelial lymphoid tissue. The cystic contents are typically white in color and may include keratinized material or cholesterol crystals. Adsay et al classified cystic lesions covered by the squamous epithelium of the pancreas as LECs as epidermoid (those occurring in the subpancreatic epithelium) or dermoid (those with cutaneous appendages).^55^

The pathological diagnostic criteria for LECs are ambiguous. Presently, the predominant diagnostic criterion of an LEC is a lumen surface with a stratified squamous epithelium and abundant lymphoid tissue underneath. The reports so far classified cysts as guided by Adsay et al^55^ : “LECs are characterized microscopically by stratified squamous epithelium surrounded by a band of mature lymphoid tissue with intervening well‐formed germinal centers.” Adsay et al also added the following: “The second type of squamous‐lined cyst in the pancreas is the epidermoid cyst arising in intrapancreatic accessory spleen.” These investigators did not mention lymphoid‐rich cysts in the accessory spleen. Some of the cases epidermoid cysts described by Truong et al in 1987 are now thought to be LECs. Owing to the unclear classification criteria, it is possible that other cases reported as epidermoid cysts are also LECs.

In the present case, the lumen epithelium of the multilocular cyst consisted of mature squamous material with developed subepithelial lymphoid tissue. In addition, white and the red pulp were detected in the pancreatic accessory spleen; hence, the cyst was diagnosed as a splenic LEC. There are three theories regarding the pathogenesis of pancreatic LECs.^6^ The first theory suggests that LECs originate in the misplaced branchial cleft tissue because of the histologic resemblance. The second suggests that squamous metaplasia in an obstructed pancreatic duct, which subsequently protrudes into a peripancreatic lymph node, gives rise to LECs. The third links LECs to cyst development from an ectopic pancreas in a peripancreatic lymph node. At present, a consensus has not been reached. Tateyama et al summarized the findings of their immunohistochemical study as follows: “The cytokeratin phenotypes of the epithelial lining of LEC were similar to those of the epithelial retention cysts but different from those of branchial cleft cysts. In addition to the cytokeratin pattern, the presence of some islets and ducts in the fibrous wall of the LEC might support the second hypothesis.” In the present case, the LEC was found in the accessory spleen in the pancreas, which is consistent with the second hypothesis. However, further consideration and discussion are required.

## 4 CONCLUSION

This study is the first report of an LEC in the intrapancreatic accessory spleen. The diagnostic criteria for LECs are ambiguous, and the difference between LECs and epidermoid cysts is unclear. It is necessary to consider LECs from a pathological point of view.

## CONFLICT OF INTEREST

None declared.

## AUTHOR CONTRIBUTIONS

SH: drafted the manuscript. MH, HN, and TI: supervised the preparation of the manuscript and reviewed and modified the manuscript. YM: contributed to the surgery and reviewed and modified the manuscript. RY: contributed to the pathological diagnosis and reviewed and modified the manuscript. TN: contributed to the pathological diagnosis and TI reviewed and modified the manuscript. All authors read and approved the final manuscript.

## ETHICAL APPROVAL

This manuscript has not been published elsewhere, and this treatment strategy has been approved by the appropriate ethics review board.

## Data Availability

This manuscript contains all the necessary data in the text.
